# IL6/IL10/TLR4 Govern Immunogenic Cell Death in Aortic Dissection

**DOI:** 10.1155/cdr/4140685

**Published:** 2026-03-28

**Authors:** Yukui Du, Dongqing Chang, Bofeng Yu, Li Zhang, Liang He, Fengxia Wang, Shaoyan Chang

**Affiliations:** ^1^ Department of Cardiac Surgery, Center for Cardiac and Panvascular Medicine, People′s Hospital of Xinjiang Uygur Autonomous Region, Urumqi, Xinjiang, China; ^2^ Department of Anesthesiology, People′s Hospital of Xinjiang Uygur Autonomous Region, Urumqi, Xinjiang, China; ^3^ Cardiovascular Medicine Department, Center for Cardiac and Panvascular Medicine, People′s Hospital of Xinjiang Uygur Autonomous Region, Urumqi, Xinjiang, China; ^4^ Capital Center for Children′s Health, Capital Medical University, Capital Institute of Pediatrics, Beijing, China, shouer.com.cn

**Keywords:** aortic dissection, biomarkers, immune infiltration, programmed cell death, regulatory networks, therapeutic targets, transcriptome sequencing

## Abstract

**Background:**

Aortic dissection (AD), a life‐threatening cardiovascular emergency, poses a significant threat to global cardiovascular health. Emerging evidence implicates programmed cell death (PCD) as a critical driver of AD pathogenesis, yet the molecular mechanisms remain poorly defined. This study systematically investigates PCD‐related biomarkers in AD to identify novel therapeutic targets.

**Methods:**

Multiomics analysis integrated transcriptome sequencing data from eight AD and five control aortic tissues. Differentially expressed genes (DEmRNAs, *n* = 3094) were intersected with 1574 programmed cell death–related genes (PCD‐RGs) to identify candidate biomarkers. Protein–protein interaction (PPI) networks, functional enrichment, immune infiltration profiling, and drug‐target prediction were employed to characterize key molecular drivers.

**Results:**

Three PCD‐associated biomarkers were identified: IL6 (11.8‐fold upregulated), IL10 (26.1‐fold upregulated), and TLR4 (0.38‐fold downregulated). These biomarkers exhibited significant enrichment in immunoregulatory pathways including “MYC Targets V1” (FDR < 0.05), with IL10 showing strong immune microenvironment correlations (memory B cells: *r* = 0.62; endothelial cells: *r* = −0.58). Regulatory network analysis revealed 58 transcription factors (STAT1/SP1/IRF8) and six key miRNAs (e.g., miR‐204‐5p) orchestrating biomarker expression. Drug‐target mapping identified 91 repurposable agents, including etanercept‐szzs (dual IL6/IL10 inhibitor) and TLR4‐modulating compounds.

**Conclusion:**

This study establishes IL6, IL10, and TLR4 as central regulators of PCD‐mediated AD pathogenesis, implicating immune–vascular crosstalk and immunogenic cell death as therapeutic entry points. The identified biomarker network and drug candidates provide a translational framework for developing precision therapies against AD.

## 1. Introduction

Aortic dissection (AD) is a critical cardiovascular disease with a high‐mortality rate, often with insidious onset. Consequently, prevention strategies are of paramount importance. Currently, there is no well‐established prevention strategy for AD, making the exploration of its pathogenesis and the identification of potential prevention targets particularly significant.

AD, a catastrophic cardiovascular emergency with mortality rates exceeding 50% within 48 h of onset [1], represents a critical unmet need in preventive cardiology. Despite advances in surgical interventions, the absence of effective prevention strategies underscores the urgency to unravel its molecular drivers [2, 3]. AD arises from multifactorial etiologies—although 20% of cases are associated with genetic disorders like Marfan syndrome [4], the majority are sporadic and linked to modifiable risk factors including hypertension (present in 72% of patients) [5], aging, and smoking [6, 7].

Central to AD pathogenesis is the progressive loss of aortic medial smooth muscle cells (SMCs), a hallmark shared across hereditary and sporadic forms [8]. This SMC depletion triggers elastin degradation, inflammatory infiltration, and ultimately, biomechanical failure of the aortic wall [9, 10]. Mounting evidence implicates dysregulated programmed cell death (PCD) as the linchpin connecting SMC homeostasis collapse to aortic wall destabilization [11]. Under physiological conditions, PCD pathways—including apoptosis, necroptosis, and pyroptosis—orchestrate tissue integrity through controlled cell turnover [12]. However, in AD, hemodynamic stress and metabolic insults induce maladaptive PCD activation, accelerating SMC loss while generating damage‐associated molecular patterns (DAMPs) that fuel sterile inflammation [13, 14].

Recent breakthroughs have expanded the PCD paradigm beyond classical apoptosis to encompass immunogenic cell death (ICD), where dying cells directly modulate immune responses [15]. In vascular contexts, ICD may drive the transition from localized SMC apoptosis to panaortic immune activation—a potential mechanism underlying AD progression [16]. Nevertheless, the specific PCD subtypes and their regulatory networks in AD remain poorly characterized, hampering the development of targeted therapies.

To address this gap, we conducted a multiomics investigation of AD aortic tissues, integrating transcriptomic profiling with computational biology approaches. Our objectives were threefold: identify programmed cell death–related gene (PCD‐RG) signatures distinguishing AD from normal aorta, decipher immune microenvironment alterations linked to PCD dysregulation, and propose druggable targets for intercepting PCD‐driven aortic wall degeneration. This study provides the first comprehensive map of PCD‐immune crosstalk in AD, revealing IL6, IL10, and TLR4 as central regulators of ICD‐mediated vascular injury. This study primarily focuses on the integration of multiomics regulatory networks (transcription factor [TF]–miRNA–lncRNA–circRNA), combined with drug repositioning analysis, to identify biomarkers and provide novel mechanistic insights into these biomarkers.

## 2. Materials and Methods

### 2.1. Subjects and Data Collection

In this study, a total of 13 samples were collected from the clinic at the Department of Cardiac Surgery, Center for Cardiac and Panvascular Medicine, People′s Hospital of Xinjiang Uygur Autonomous Region, including five control tissue samples and eight AD tissue samples. Notably, the study was approved by the Ethics Committee of People′s Hospital of Xinjiang Autonomous Region, and informed consent was obtained from all participants.

Furthermore, a total of 1574 unique PCD‐RGs were identified by combining and deduplicating genes from various sources: 1548 PCD‐RGs from 18 studies [13], 23 disulfidptosis‐related genes [15], and 39 mitochondrial permeability transition (MPT)–related genes [17] (Table S1).

### 2.2. Transcriptomic Profiling and Preprocessing

Total RNA was extracted using TRIzol (Invitrogen) and quality‐controlled via NanoDrop ND‐1000 (OD260/280 > 1.8) and Bioanalyzer 2100 (RIN > 7.0). Poly(A)–enriched RNA libraries were prepared using the NEBNext Ultra II RNA Library Prep Kit (NEB), followed by paired‐end sequencing (PE150) on the Illumina NovaSeq 6000 platform. Raw reads were preprocessed using Fastp (v0.23.2) to remove adapters and low‐quality bases (Q20 < 90*%*). Clean reads were aligned to the GRCh38 reference genome via HISAT2 (v2.2.1), with transcript quantification performed using featureCounts.

### 2.3. Differential Expression Analysis

Differentially expressed mRNA genes (DEmRNAs), differentially expressed miRNAs (DE‐miRNAs), differentially expressed circRNAs (DE‐circRNAs), and differentially expressed lncRNAs (DE‐lncRNAs) were identified using DESeq2 (v1.38.0) [17], applying thresholds of |log2FC| > 1 and Benjamini–Hochberg adjusted *p* < 0.05. Volcano plots (ggplot2 v3.4.4) and heatmaps (ComplexHeatmap v2.14.0) visualized the Top 10 up/downregulated features ranked by fold change.

### 2.4. Functional Analysis of Candidate Genes

Intersection of DEmRNAs with PCD‐RGs (VennDiagram v1.7.3) yielded 306 candidate genes. Gene Ontology (GO) and Kyoto Encyclopedia of Genes and Genomes (KEGG) enrichment analyses were performed using clusterProfiler (v4.7.1.003)21 (FDR < 0.05). Protein–protein interaction (PPI) networks were constructed via STRING (confidence score > 0.4) and visualized in Cytoscape (v3.9.1). Hub genes were prioritized using CytoHubba′s Degree and Maximal Clique Centrality (MCC) algorithms.

### 2.5. Biomarker Identification and Mechanistic Validation

Candidate biomarkers were validated via Wilcoxon rank‐sum test (*p* < 0.05). Gene set enrichment analysis (GSEA) utilized ranked Spearman correlations (psych v2.2.9) against the Hallmark gene set (MSigDB h.all.v2023.2). Functional interactomes were mapped using GeneMANIA with default parameters.

### 2.6. Immune Microenvironment Characterization

Single‐sample gene set enrichment analysis (ssGSEA) (GSVA v1.46.0) [18] quantified infiltration scores for 30 immune/stromal cell types. Differentially infiltrated populations (*p* < 0.05, Wilcoxon test) were correlated with biomarker expression (|*S* *p* *e* *a* *r* *m* *a* *n* ^′^ *s* *ρ*| > 0.30, *p* < 0.05).

### 2.7. Genomic and Regulatory Network Mapping

Chromosomal loci were annotated using RCircos (v1.2.2). TF–biomarker interactions were predicted via NetworkAnalyst, whereas miRNA targets were identified using miRanda (multiMiR v1.20.0)27. Competitive endogenous RNA (ceRNA) networks integrating DE‐lncRNAs (StarBase) and DE‐circRNAs (|*ρ*| > 0.50, *p* < 0.05) were reconstructed in Cytoscape.

### 2.8. Therapeutic Target Prioritization

Drug–gene interactions were curated from DGIdb, and ICD scores were derived via ssGSEA of ICD‐related genes (|*ρ*| > 0.30, corrplot v0.92).

### 2.9. Statistical Analysis

Statistical analysis was conducted using R (v4.2.2). Differences between the two groups were assessed using the Wilcoxon test (*p* < 0.05).

## 3. Results

### 3.1. Identification of Differentially Expressed Molecular Profiles

Multiomics differential expression analysis systematically identified four categories of significantly dysregulated noncoding RNAs and protein‐coding genes in AD samples: (1) 3094 differentially expressed genes (DEmRNAs; 2198 upregulated/896 downregulated), (2) 275 differentially expressed miRNAs (DE‐miRNAs; 138 upregulated/137 downregulated), (3) 32 uniformly upregulated circRNAs (DE‐circRNAs), and (4) 890 DE‐lncRNAs (606 upregulated/284 downregulated) (Figures 1a, 1b, 1c, 1d, and 1e). These molecular profiles established a critical foundation for subsequent mechanistic investigations.

Figure 1Transcriptome sequencing results of aorta samples from AD patients and heart controls. (a) Flowchart of transcriptomic sequencing process for aortic samples from AD patients and controls. (b) The differentially expressed gene in aortic samples from AD patients and controls. (c) The differentially miRNAs in aortic samples from AD patients and controls. (d) The differentially circRNAs in aortic samples from AD patients and controls. (e) The differentially lncRNAs in aortic samples from AD patients and controls.

**Figure:**
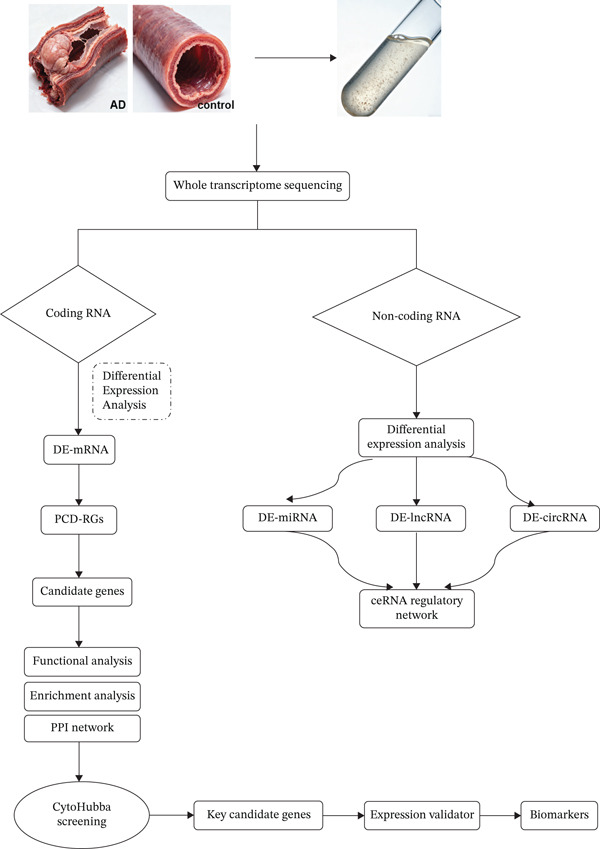
(a)

**Figure:**
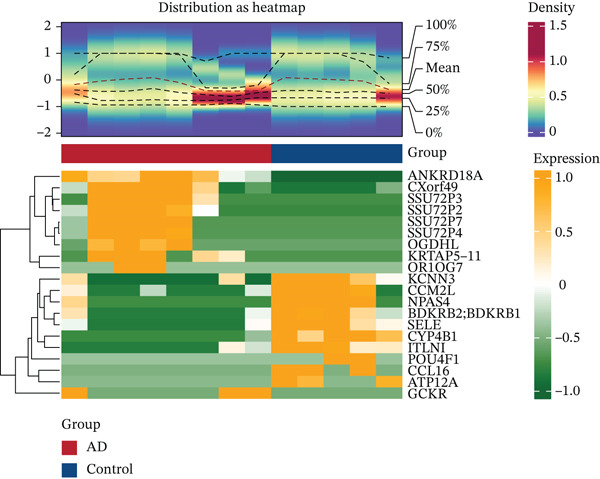
(b)

**Figure:**
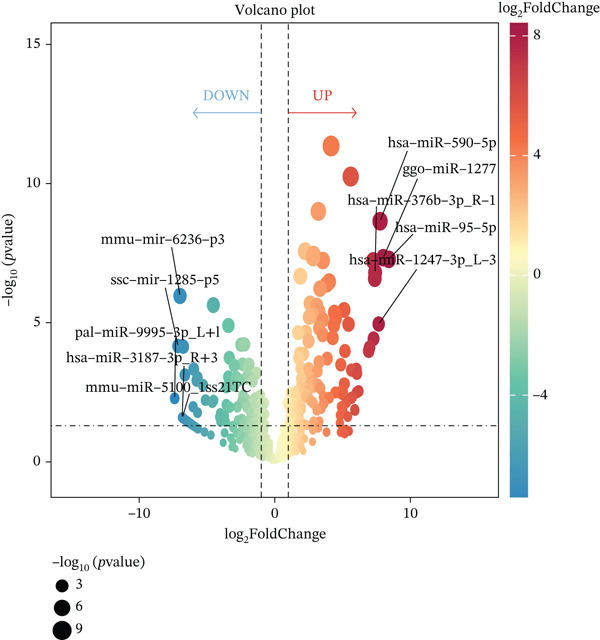
(c)

**Figure:**
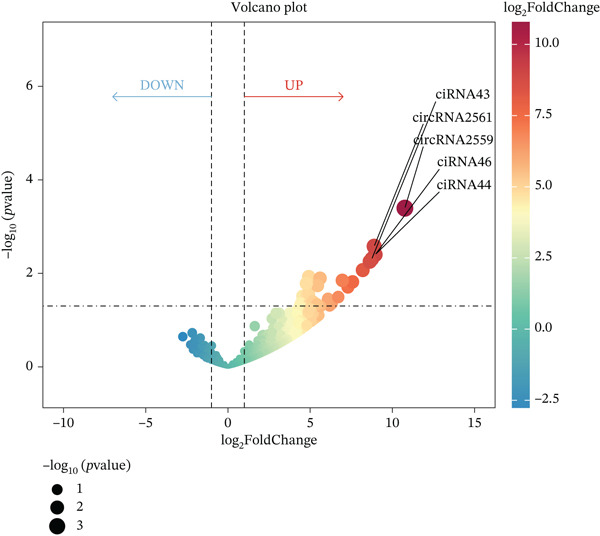
(d)

**Figure:**
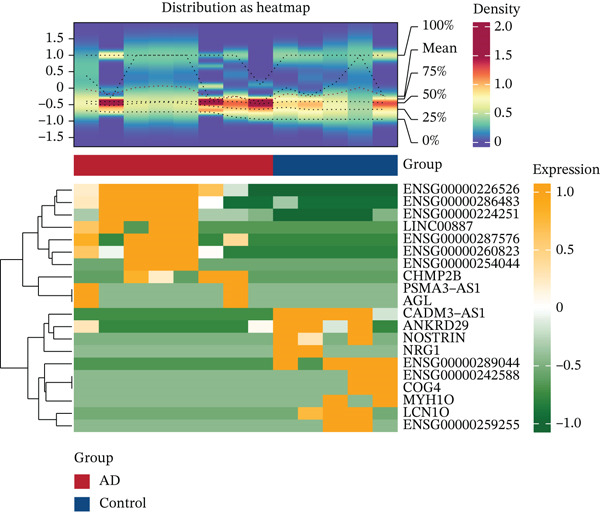
(e)

### 3.2. Functional Characterization of Candidate Genes and Biomarker Screening

Integration of 3094 DEmRNAs with 1574 PCD‐RGs yielded 306 functional candidate genes (Figure 2a). Multidimensional functional annotation revealed significant enrichment in 2830 GO terms, including “regulation of apoptotic signaling pathway” (BP), “vacuolar membrane” (CC), and “protein serine/threonine kinase activity” (MF), along with 141 KEGG pathways such as PI3K‐Akt signaling, apoptosis, and proteoglycans in cancer (Figure 2b,c and Tables S2 and S3). Topological analysis of the PPI network identified a core regulatory module comprising 185 nodes and 309 interactions, with IL6 demonstrating critical interactions with IL10 and STAT3 (Figure 2d). Hub genes were comprehensively evaluated using two algorithms, CytoHubba′s degree and MCC, to assess their global connectivity and local centrality, respectively. Ultimately, IL6, IL10, and TLR4 were identified as core biomarkers (Figure 2e,f).

Figure 2Results of gene set analysis of differentially expressed genes and CDD‐RGS in AD patients compared with normal aorta samples. (a) The Venn diagram analysis results of 3094 DEmRNAs and 1574 PCD‐RGs showed that 306 genes intersected between them. (b) GO analysis results of 306 candidate genes. (c) KEGG analysis results of 306 candidate genes. (d) According to the results of enrichment analysis, 121 abnormal genes were removed, and the remaining 185 genes were analyzed for PPI, showing 309 interacting PPI networks. (e) 185 genes in the PPI network were scored to show the Top 10 genes with the highest connectivity in each algorithm. (f) Three candidate biomarkers (IL6, IL10, and TLR4) were identified by joint analysis of MCC and degree.

**Figure:**
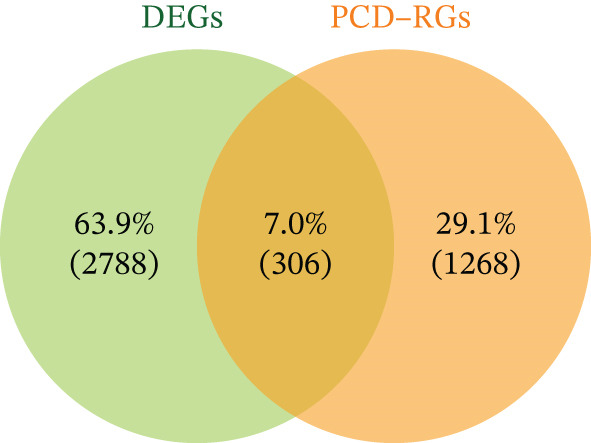
(a)

**Figure:**
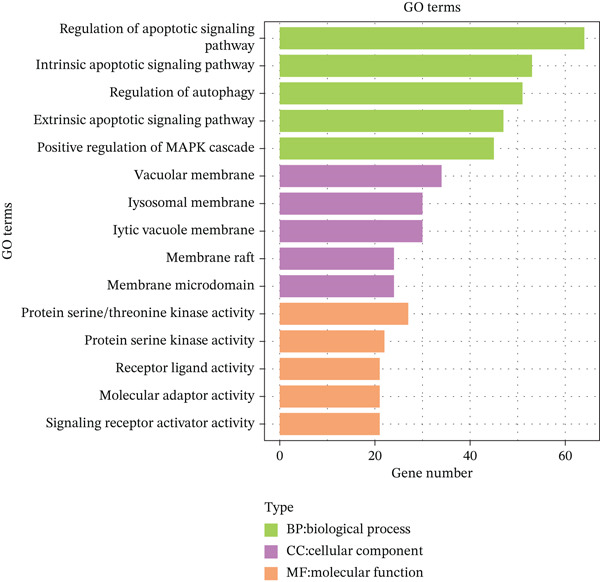
(b)

**Figure:**
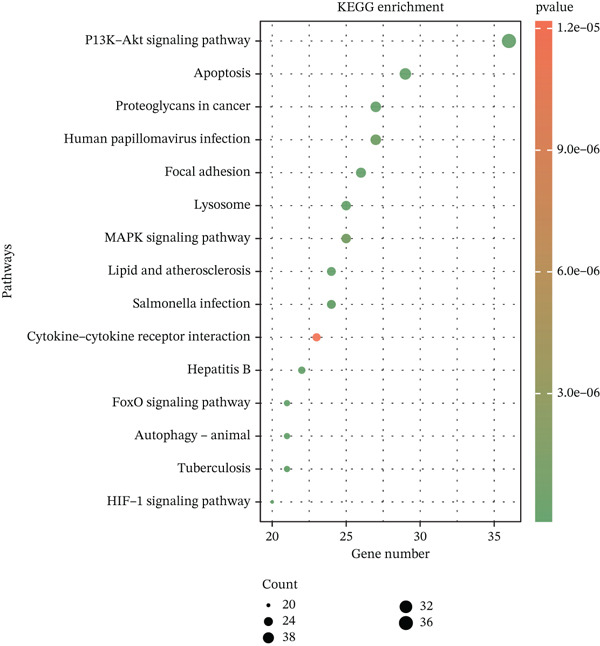
(c)

**Figure:**
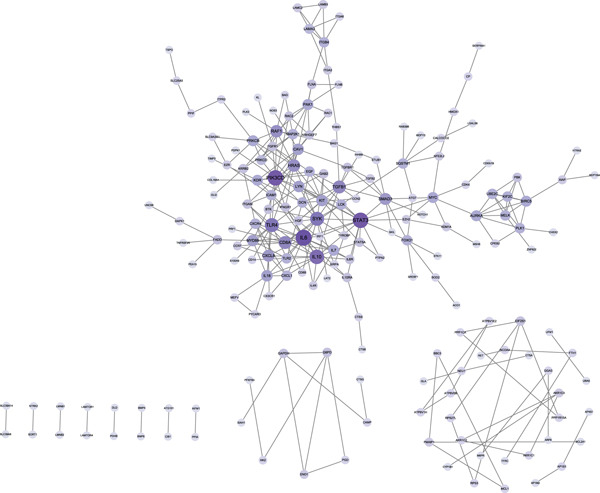
(d)

**Figure:**
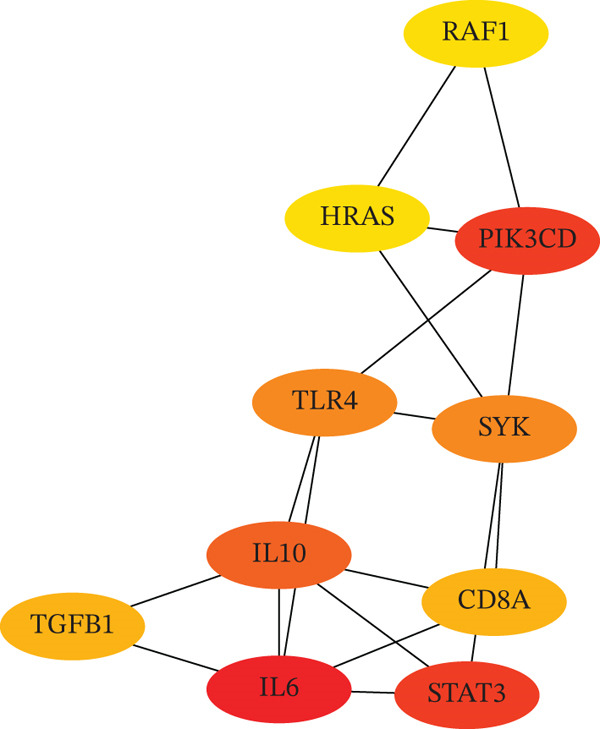
(e)

**Figure:**
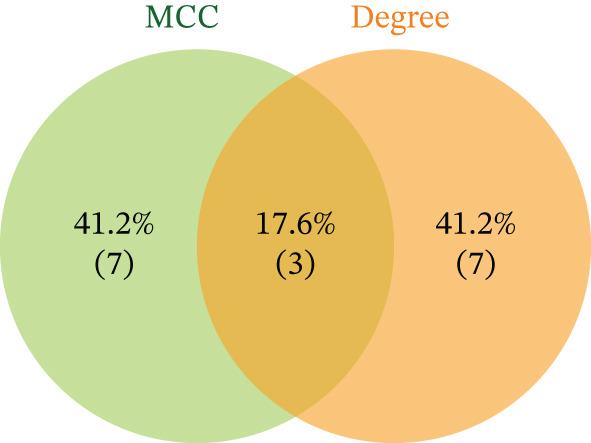
(f)

### 3.3. Functional Validation and Interaction Networks of Biomarkers

Quantitative analysis demonstrated significant dysregulation of the key hub genes in AD samples. Specifically, IL6 and IL10 were markedly upregulated, exhibiting 11.8‐fold and 26.1‐fold increases, respectively, whereas TLR4 expression was reduced to 38% of control levels (Figure 3a). To assess the robustness of these findings, we analyzed the expression patterns of IL6, IL10, and TLR4 in an independent public transcriptomic dataset (GSE52093), also derived from AD studies. Although these expression differences did not reach statistical significance in the external cohort, the directional trends for all three genes were consistent with those observed in our own findings (Figure S1), thereby supporting their potential biological relevance in AD pathogenesis. Furthermore, GSEA indicated that IL6 and IL10 are coregulated within the “TNFA signaling via NFKB,” whereas IL10 and TLR4 are jointly involved in the “MYC targets V1” pathways (Figure 3b). Interaction network analysis identified key functional pairs including IL10‐LY96, IL6‐TLR6, and TLR4‐TIRAP, predominantly associated with “response to bacterial‐derived molecules” (Figure 3c and Table S4).

Figure 3Expression and function analysis of three biomarkers IL6, IL10, and TLR4 in AD patients. (a) Expression results of three biomarkers, IL6, IL10, and TLR4 in aorta samples from AD patients; (b) the pathways involved in the three candidate genes, IL6, IL10, and TLR4 were analyzed by GSEA; and (c) the interaction pathway between the three biomarkers and 20 interacting functional genes was revealed by GeneMANIA analysis.

**Figure:**
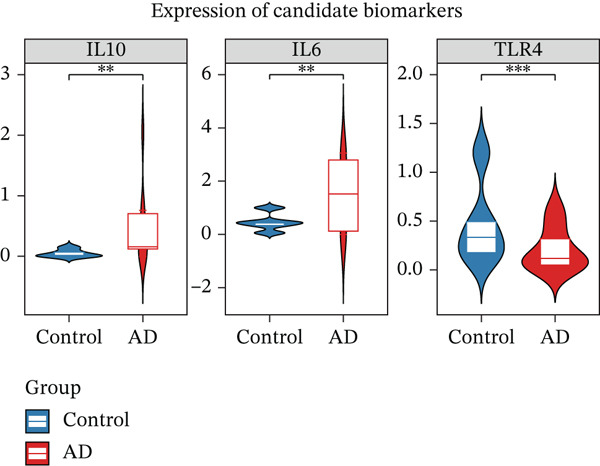
(a)

**Figure:**
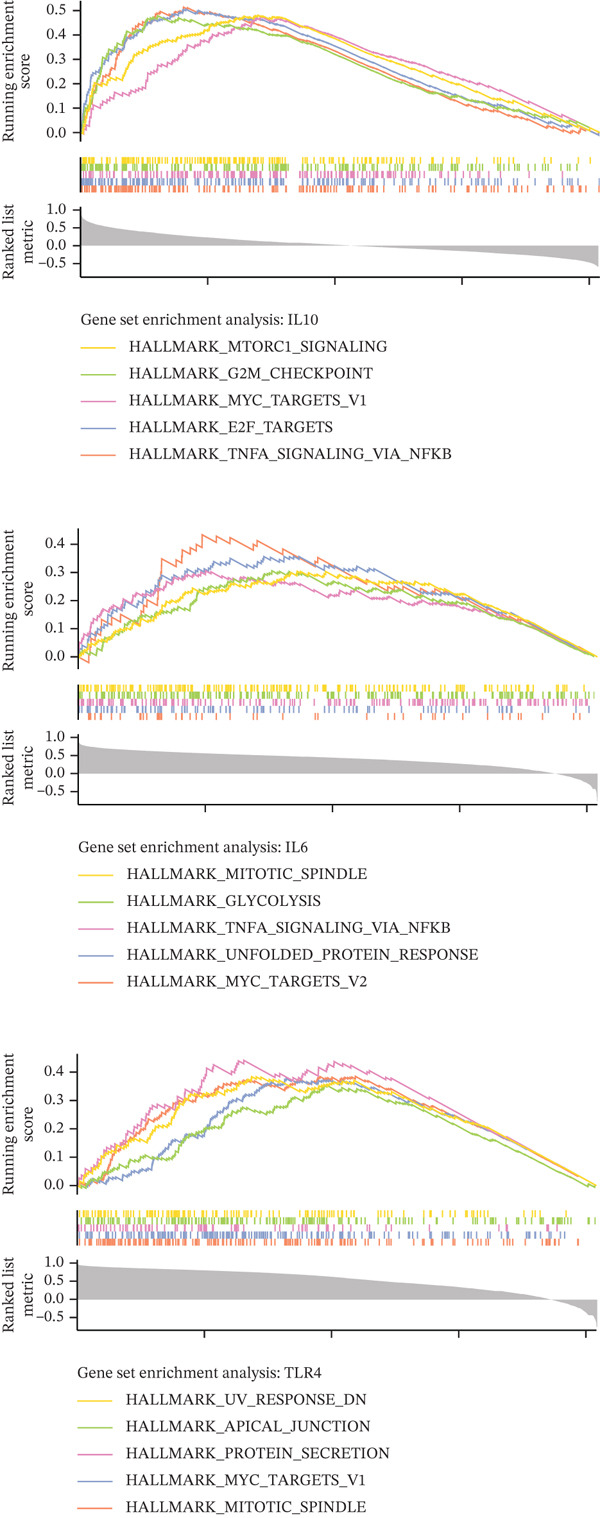
(b)

**Figure:**
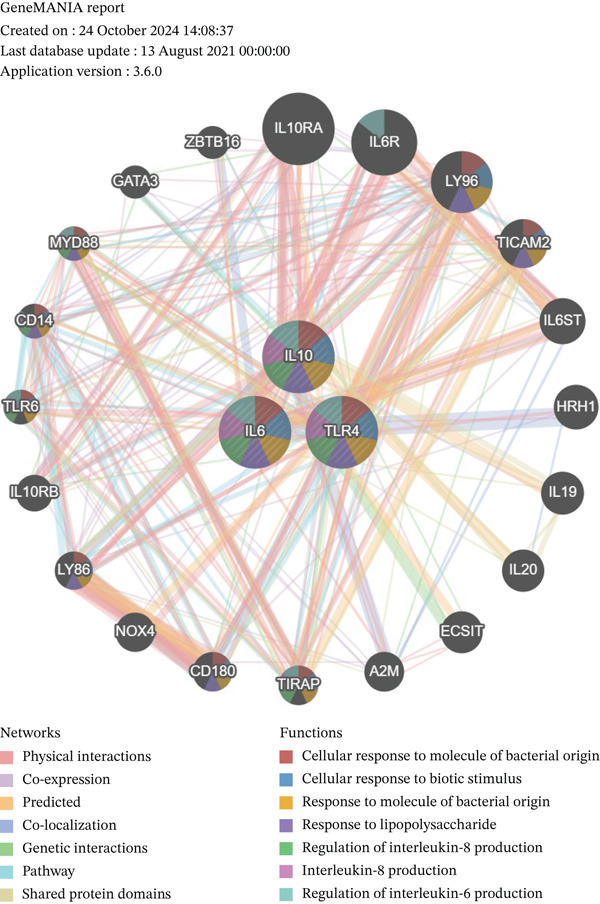
(c)

### 3.4. Immune Microenvironment Features and Genomic Localization

To investigate immune cell infiltration in AD and control samples based on transcriptome sequencing data, we performed immune infiltration analysis using the ssGSEA algorithm. This method enabled us to quantify infiltration scores for 28 distinct immune cell types, as well as for two stromal cell types: fibroblasts and endothelial cells. Differences in infiltration scores between AD patients and control samples were evaluated using the Wilcoxon rank‐sum test. The results revealed significant differences in the infiltration scores of five immune cell types between AD patients and control subjects (Figure 4a). Specifically, immune infiltration profiling showed significantly increased memory B cell infiltration in AD samples (*p* < 0.05), whereas effector memory CD8+ T cells, NK cells, endothelial cells, and fibroblasts predominated in controls. Furthermore, a correlation analysis was conducted between the three candidate biomarkers′ expression levels and the infiltration scores of various immune cell subsets. The results demonstrated a strong positive correlation between IL10 and memory B‐cell infiltration (*r* = 0.62, *p* < 0.05) and significant negative correlation with endothelial cells (*r* = −0.58, *p* < 0.05) (Figure 4b). Chromosomal localization analysis mapped IL10 to 1q32, IL6 to 7p15, and TLR4 to 9q33, suggesting potential genomic influences on their AD‐related functions (Figure 4c).

Figure 4Immunoinfiltration analysis and chromosome localization analysis of the three candidate biomarkers. (a) The comparison results of 30 immune/stromal cells infiltration scores between AD patients and the control group. The ssGSEA method was used to calculate immune cell infiltration in different groups, and it was found that 28 distinct immune cell types and 2 stromal cell types were involved in the immune infiltration process. Results from Wilcoxon statistical analysis revealed significant differences in five kinds of immune cell infiltration scores between AD patients and control group, including memory B cells with higher infiltration levels in AD samples, as well as effector memory CD8+ T cells, natural killer (NK) cells, endothelial cells, and fibroblasts that showed higher infiltration levels in the control group; (b) correlation analysis results of five kinds of immune cells with the candidate biomarkers. Correlation analysis revealed strong positive association between IL10 and memory B cells (*r* = 0.62, *p* < 0.05), and significant negative correlation with endothelial cells (*r* = −0.58, *p* < 0.05), and (c) a diagram showing the specific chromosomal localization of the three biomarkers (IL10, IL6, and TLR4).

**Figure:**
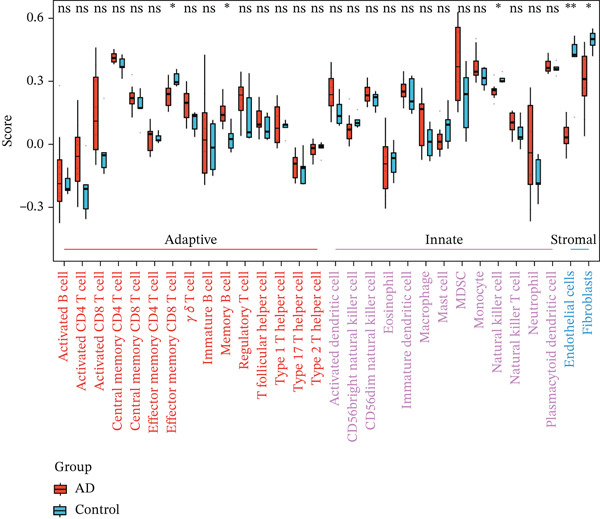
(a)

**Figure:**
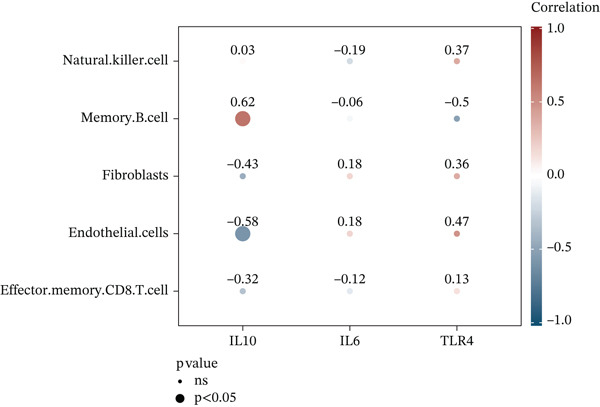
(b)

**Figure:**
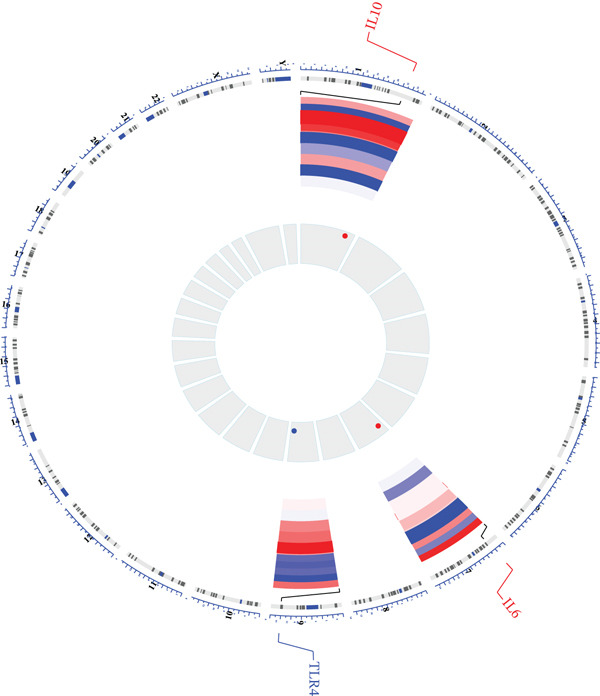
(c)

### 3.5. Multilayered Regulatory Network Construction

Integrated analysis of transcriptional regulation and ceRNA mechanisms revealed coregulation of IL6/IL10 by STAT1/SP1 TFs and IRF8‐mediated targeting of TLR4/IL10 (Figure 5a). Additionally, we identfied six key miRNAs (e.g., hsa‐miR‐548e‐3p) targeting biomarkers (Figure 5b,c), as well as specific TF‐miRNA regulatory axes, including MSC‐IL10‐hsa‐miR‐204‐5p (Figure 5d). Five key lncRNAs (PSMA3‐AS1, LINC01278, etc.) modulated IL10 via miR‐381‐3p/miR‐204‐5p sponging (Figure 5e,f). Five circRNAs (e.g., hsa‐circ‐0007179) showed strong positive correlations with miR‐381‐3p (|*r*| > 0.50, *p* < 0.05) (Figure 5g), ultimately forming a multidimensional ceRNA network (Figure 5h,i).

Figure 5Analysis of the potential regulatory mechanism of three biomarkers. (a) The transcription factors for IL6, IL10, and TLR4 were predicted by NetworkAnalyst; (b) Venn diagram results of predicted miRNAs (duplicate removal) and differential miRNAs using miRanda database; (c) six miRNAs in the Wayne diagram target the results of biomarkers, among which four miRNAs (HSA‐mir‐548e‐3p, HSA‐mir‐204‐5p, hsa‐miR‐597‐5p, and hsa‐miR‐381‐3p) target IL10, whereas two miRNAs (hsa‐RNA) target IL10. (d) Creating a miRNA network of transcription factors and biomarkers. (e) Venn diagram of 210 lncRNAs targeting miRNAs and 890 differential lncRNAs. (f) A key lncRNAs‐miRNAs–biomarkers network is constructed. (g) Carrying out correlation analysis on two miRNAs in the network of Figure (f), and evaluating their relationship with differential cirRNAs. (h) The circRNA/lncRNA–miRNA–biomarker network is constructed. (i) Sangji diagram shows the potential interaction among transcription factors (left column), candidate genes (middle column), miRNAs (right column), and lncRNAs (right second column). The different colors of each column represents different molecular entities, and the ribbon streamline connecting these color blocks represents the predicted interaction path; the width of streamline corresponds to the strength of interaction or the confidence of prediction.

**Figure:**
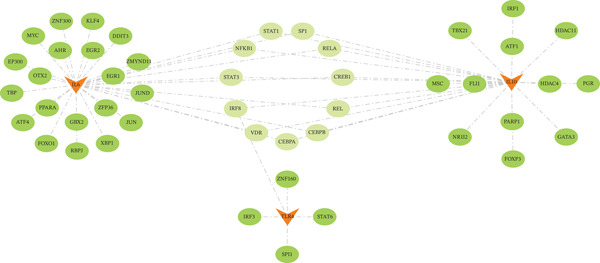
(a)

**Figure:**
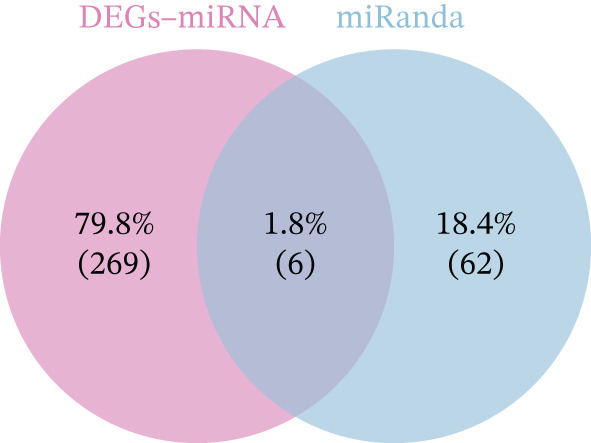
(b)

**Figure:**
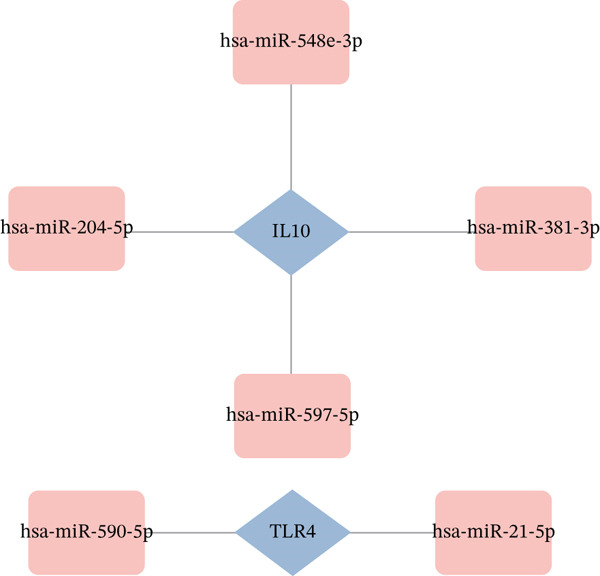
(c)

**Figure:**
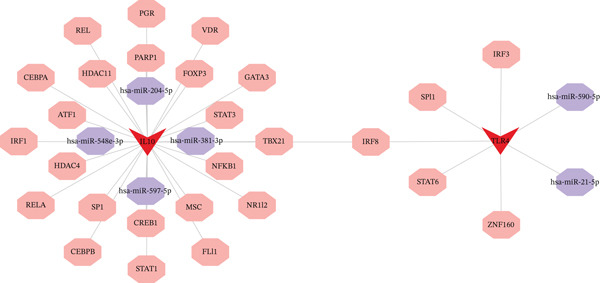
(d)

**Figure:**
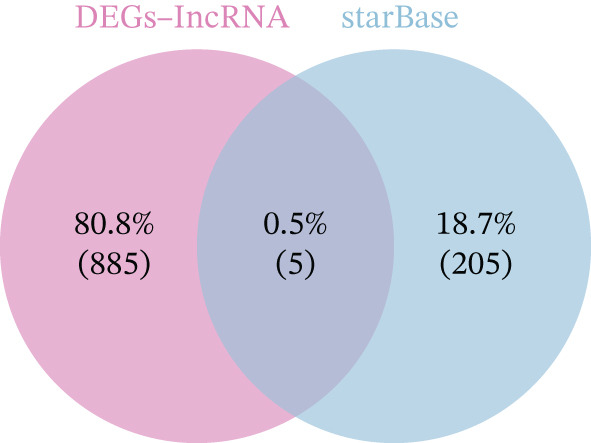
(e)

**Figure:**
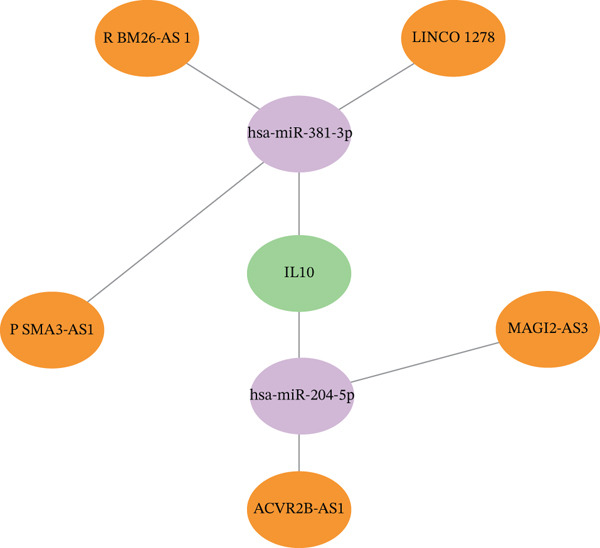
(f)

**Figure:**
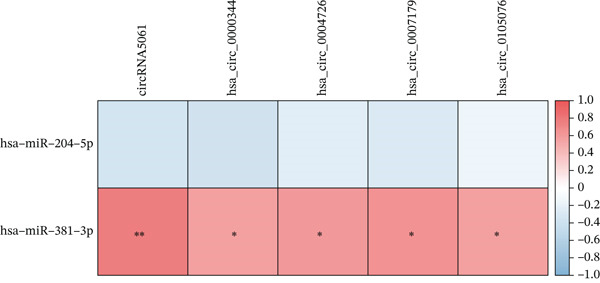
(g)

**Figure:**
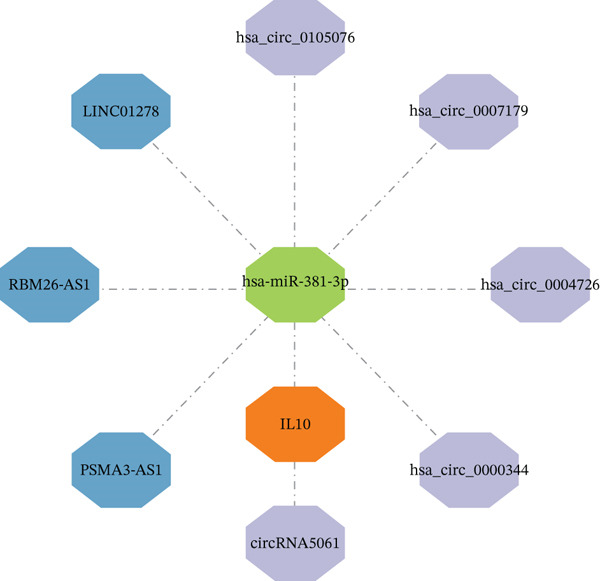
(h)

**Figure:**
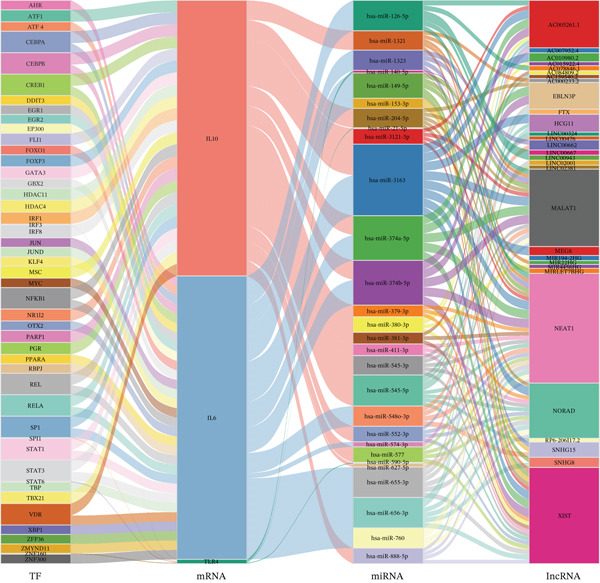
(i)

### 3.6. Therapeutic Targets and ICD Mechanisms

Drug database mining identified 48 IL6‐targeting agents (e.g., infliximab‐dyyb), 20 IL10‐related compounds, and 23 TLR4 modulators, with etanercept‐szzs showing dual targeting of IL6/IL10 (Figure 6a). Mechanistically, IL6/IL10/TLR4 were implicated in ICD‐mediated AD pathogenesis: AD samples exhibited significantly elevated ssGSEA scores for ICD‐related genes (*p* = 0.0031), with TLR4 showing strong positive correlation with HMGB1 (*r* = 0.88, *p* < 0.05) (Figure 6b,c), suggesting ICD‐mediated promotion of aortic medial degeneration and dissection progression.

Figure 6The role of biomarkers in the pathogenesis of AD and predicts the targeted drugs. (a) Using DGIdb database to search for drugs targeting three biomarkers; (b) IL‐6, IL‐10, and TLR4 are involved in immunogenic cell death (ICD), and ssGSEA scores of AD and control samples are calculated by using ICD‐related genes. (c) Correlation analysis results of IL‐6, IL‐10, and TLR4 with ICD‐related genes.

**Figure:**
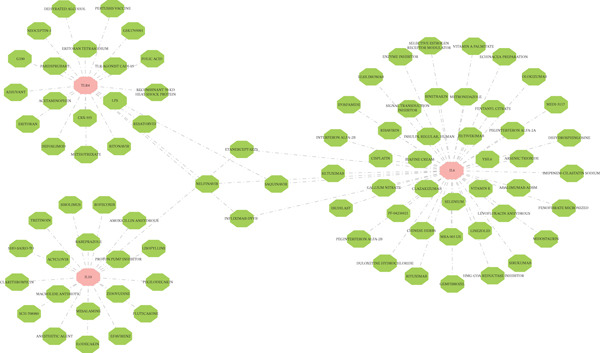
(a)

**Figure:**
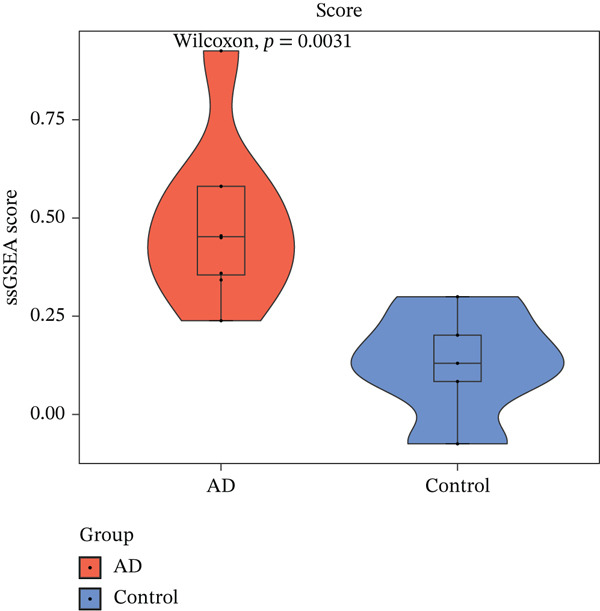
(b)

**Figure:**
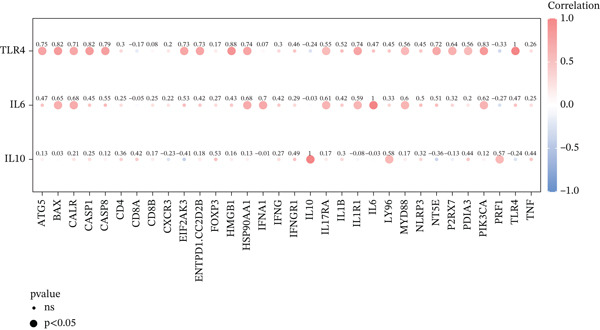
(c)

These findings establish IL6, IL10, and TLR4 as a diagnostic triad for AD, mechanistically linked to immune dysregulation, vascular remodeling, and ICD‐driven medial degeneration. The identified ceRNA networks and drug‐target interactions provide a roadmap for biomarker‐driven therapeutics and personalized AD management.

## 4. Discussion

This multiomics study advances our understanding of AD pathogenesis by identifying IL6, IL10, and TLR4 as central regulators of ICD‐mediated vascular remodeling. The transcriptional dysregulation observed—IL6 (11.8↑), IL10 (26.1↑), and TLR4 (0.38↓)—aligns with the established paradigm where mechanical stress disrupts vascular smooth muscle cell (vSMC) homeostasis, driving phenotypic switching, and aortic wall degeneration [2, 8, 19]. Our findings extend this model by implicating ICD as a novel mechanism linking immune dysregulation to medial layer injury.

### 4.1. IL6‐Driven Ferroptosis in AD Pathogenesis

IL‐6 was significantly upregulated (11.8‐fold), supporting its dual role as both an inflammatory mediator and a ferroptosis inducer [20]. IL6 may exacerbate endoplasmic reticulum stress via iron overload via the JAK‐STAT signaling pathway [21], forming a positive feedback loop that promotes vSMC apoptosis, a critical driver of aortic medial degeneration [8, 22]. These findings are consistent with recent evidence that IL6 acts as a major regulator of matrix metalloproteinase activation in aortic aneurysms [23]. Notably, IL6′s prosurvival functions in cancer [24] contrast with its proapoptotic role observed here, suggesting tissue‐specific signaling outcomes.

### 4.2. The IL10 Paradox: Anti‐Inflammatory Sentinel or Covert Pathogenic Driver?

Although IL10 is canonically anti‐inflammatory [18, 19, 25, 26], its dramatic overexpression (26.1‐fold) in AD samples suggests context‐dependent roles. We propose two nonmutually exclusive mechanisms: Compensatory suppression: counteracting IL6‐driven inflammation through STAT3‐mediated inhibition of NF‐*κ*B [27] and immune dysregulation: disrupting Treg/Th17 balance, as observed in IL10‐overexpressing abdominal aortic aneurysms [28]. A strong correlation between IL‐10 and memory B cells (*r* = 0.62) is consistent with findings reported in autoimmune vasculitis, suggesting that a compensatory anti‐inflammatory response is occurring in the body [25]. B cells limit excessive T cell activation and macrophage inflammation by secreting IL‐10, thereby attenuating vascular wall injury. In contrast, IL‐10 is negatively correlated with endothelial cells (*r* = −0.58). Although previous studies have demonstrated that IL‐10 generally exerts a protective effect on endothelial cells, severe inflammatory responses under profound pathological conditions lead to reactive elevation of IL‐10 and aggravated endothelial damage with impaired cellular barrier function [29]. In addition, endothelial‐to‐mesenchymal transition may occur, which is also one of the key pathogenic mechanisms in atherosclerosis [19].

### 4.3. TLR4 Downregulation: Protective Adaptation or Maladaptive Response?

Contrary to its canonical proinflammatory role [30–33], significant downregulation of TLR4 (0.38‐fold) in AD presents a complex biological paradox. Although inhibition of TLR4 is generally thought to alleviate macrophage‐mediated inflammatory injury [32], our data suggest its downregulation may impair efferocytosis, a process critical for the timely clearance of necrotic cells and resolution of vascular inflammation [34]. This paradoxical observation is consistent with findings in Tyrobp‐modified AD mouse models, in which TLR4 knockdown unexpectedly accelerated medial necrosis via defective apoptotic cell clearance [34]. Furthermore, the involvement of TLR4 in “MYC Targets V1” pathway further links it to dysregulated proliferation of vSMC [35].

### 4.4. ICD: Bridging Cell Death and Immune Activation

In AD, aortic tearing leads to massive exposure of cryptic autoantigens in the vascular wall, including extracellular matrix components and heat shock proteins. The enrichment of memory B cells indicates that the host has established adaptive immune memory against these neoantigens. Crosstalk among IL‐6, IL‐10, and TLR4 in the ICD pathway (Figure 6b,c) reveals a critical immune‐converting mechanism: ICD drives the aortic microenvironment from an immunologically quiescent “cold state” to a hyperactive “hot state” [16, 36–38]. This process well explains the autoimmune‐like features observed in AD, including the production of antielastin autoantibodies [19]. Our ssGSEA results (*p* = 0.0031) and strong correlation between TLR4 and HMGB1 (*r* = 0.88) further confirm that ICD amplifies DAMP signaling, promoting a vicious cycle between inflammation and matrix degradation that ultimately accelerates aortic wall destruction and dissection progression.

### 4.5. Therapeutic Implications and Future Directions

The identified drug targets in the study—particularly etanercept‐szzs (IL6/IL10 inhibitor) and TLR4 modulators—offer immediate translational potential. However, the paradoxical biological effects of IL10 and TLR4 highlight the need for personalized therapeutic strategies that consider the specific pathological stage and microenvironmental context. Furthermore, the ceRNA network (e.g., hsa‐circ‐0007179/miR‐381‐3p/IL10 axis) provides a blueprint for RNA‐targeted interventions in AD.

### 4.6. Study Limitations

Although this study offers valuable insights, it has several limitations. First, the sample size (*n* = 8), although comparable with other exploratory studies utilizing omics analyses of rare clinical samples, remains small and necessitates validation in large cohorts. Second, the RNA samples have been exhausted, preventing the validation of key biomarkers (IL6, IL10, and TLR4) at the protein and functional levels; this limitation must be addressed in independent cohorts. Third, bulk sequencing does not resolve cell type–specific expression patterns, leaving the precise roles of molecules such as IL10 in the phenotypic transformation of vSMCs unclear. To address these limitations, our future research will focus on (1) immunohistochemical validation in independent AD tissue cohorts, (2) spatial transcriptomics to assess spatial expression heterogeneity, and (3) functional experiments utilizing CRISPR‐edited AD′s disease organoids or vSMCs to elucidate the mechanisms regulating PCD and immune responses in vitro.

## 5. Conclusion

This study systematically delineates the molecular landscape of AD through multiomics profiling, identifying IL6, IL10, and TLR4 as central biomarkers mechanistically linked to immune dysregulation, vascular remodeling, and ICD. The dysregulation of these biomarkers, coordinated by a multilayer regulatory network involving TFs (STAT1/SP1/IRF8), noncoding RNAs (miR‐204‐5p, miR‐381‐3p, PSMA3‐AS1, and hsa‐circ‐0007179), and ICD‐related pathways, correlates with distinct immune microenvironment alterations, particularly memory B cell accumulation and endothelial dysfunction. Drug‐target mapping reveals repurposable therapeutic agents (etanercept‐szzs and infliximab‐dyyb) while highlighting ICD modulation as a novel intervention strategy. These findings not only establish a diagnostic triad for AD but also provide a translational framework for developing biomarker‐guided therapies targeting the immune–vascular axis in aortic pathologies.

## Author Contributions

Yukui Du: conception and design of the study; Shaoyan Chang, Dongqing Chang, Li Zhang, Liang He, Fengxia Wang, and Bofeng Yu: acquisition of data or analysis and interpretation of data; Shaoyan Chang: writing—original draft preparation; Shaoyan Chang and Yukui Du: supervision and writing—reviewing and editing.

## Funding

This study was supported by the National Natural Science Fund of China (82001555/0417); Xinjiang Uygur Autonomous Region Natural Science Fund (2024D01C107); and Department of Human Resources and Social Security of Xinjiang Uygur Autonomous Region–“Tianchi Talent” Introduction Plan.

## Disclosure

Shaoyan Chang and Yukui Du had primary responsibility for the final content, and all authors read and approved the final manuscript.

## Ethics Statement

The study was conducted in accordance with the Declaration of Helsinki, and the protocol was approved by the ethics committee of People′s Hospital of Xinjiang Autonomous Region.

## Conflicts of Interest

The authors declare no conflicts of interest.

## Supporting Information

Additional supporting information can be found online in the Supporting Information section.

## Supporting information


**Supporting Information 1** Table S1: The list of programmed cell death–related genes (PCD‐RGs).

**Supporting Information 2** Table S2: GO analysis results of 2830 candidate genes.

**Supporting Information 3** Table S3: KEGG analysis results of 2830 candidate genes.

**Supporting Information 4** Table S4: Interaction network derived from KEGG and GSEA analyses of IL6, IL10, and TLR4.

**Supporting Information 5** Figure S1: The expression analysis of IL6, IL10, and TLR4 in AD and controls in an independent public transcriptomic dataset (GSE52093).

## Data Availability

The data that support the findings of this study are openly available in GEO at https://www.ncbi.nlm.nih.gov/gds (Reference Numbers GSE294606 and GSE294607).

## References

[1] Roman M. J. and Devereux R. B. , Aortic Dissection Risk in Marfan Syndrome, Journal of the American College of Cardiology. (2020) 75, no. 8, 854–856, 10.1016/j.jacc.2019.12.042, 32130919. 32130919 PMC8208625

[2] Nienaber C. A. , Clough R. E. , Sakalihasan N. , Suzuki T. , Gibbs R. , Mussa F. , Jenkins M. P. , Thompson M. M. , Evangelista A. , Yeh J. S. M. , Cheshire N. , Rosendahl U. , and Pepper J. , Aortic Dissection, Nature Reviews Disease Primers. (2016) 2, no. 1, 16053, 10.1038/nrdp.2016.53, 2-s2.0-85006233516. 27440162

[3] Yu W. M. , Chen Y. P. , Cheng A. L. , Zheng Z. Y. , Wang J. W. , Liu X. B. , and Zhou J. X. , Machine Learning-Driven Prediction Models and Mechanistic Insights Into Cardiovascular Diseases: Deciphering the Environmental Endocrine Disruptors Nexus, Journal of Translational Medicine. (2025) 23, no. 1, 10.1186/s12967-025-07223-6, 41225610. PMC12613883 41225610

[4] Tian C. , Chen Y. , Xu B. , Tan X. , and Zhu Z. , Association of Triglyceride-Glucose Index With the Risk of Incident Aortic Dissection and Aneurysm: A Large-Scale Prospective Cohort Study in UK Biobank, Cardiovascular Diabetology. (2024) 23, no. 1, 10.1186/s12933-024-02385-x, 39095822. PMC11297767 39095822

[5] Koba A. , Yamagishi K. , Sairenchi T. , Noda H. , Irie F. , Takizawa N. , Tomizawa T. , Iso H. , and Ota H. , Risk Factors for Mortality From Aortic Aneurysm and Dissection: Results From a 26-Year Follow-Up of a Community-Based Population, Journal of the American Heart Association. (2023) 12, no. 8, e027045, 10.1161/JAHA.122.027045, 37042285. 37042285 PMC10227264

[6] Chakraborty A. , Li Y. , Zhang C. , Li Y. , Rebello K. R. , Li S. , Xu S. , Vasquez H. G. , Zhang L. , Luo W. , Wang G. , Chen K. , Coselli J. S. , LeMaire S. A. , and Shen Y. H. , Epigenetic Induction of Smooth Muscle Cell Phenotypic Alterations in Aortic Aneurysms and Dissections, Circulation. (2023) 148, no. 12, 959–977, 10.1161/CIRCULATIONAHA.123.063332, 37555319. 37555319 PMC10529114

[7] Carbone A. , Ranieri B. , Castaldo R. , Franzese M. , Rega S. , Cittadini A. , Czerny M. , and Bossone E. , Sex Differences in Type A Acute Aortic Dissection: A Systematic Review and Meta-Analysis, European Journal of Preventive Cardiology. (2023) 30, no. 11, 1074–1089, 10.1093/eurjpc/zwad009, 36629802. 36629802

[8] Rombouts K. B. , van Merrienboer T. A. R. , Ket J. C. F. , Bogunovic N. , van der Velden J. , and Yeung K. K. , The Role of Vascular Smooth Muscle Cells in the Development of Aortic Aneurysms and Dissections, European Journal of Clinical Investigation. (2022) 52, no. 4, e13697, 10.1111/eci.13697, 34698377. 34698377 PMC9285394

[9] Yuan J. and Ofengeim D. , A Guide to Cell Death Pathways, Nature Reviews Molecular Cell Biology. (2024) 25, no. 5, 379–395, 10.1038/s41580-023-00689-6. 38110635

[10] Bedoui S. , Herold M. J. , and Strasser A. , Emerging Connectivity of Programmed Cell Death Pathways and Its Physiological Implications, Nature Reviews Molecular Cell Biology. (2020) 21, no. 11, 678–695, 10.1038/s41580-020-0270-8, 32873928. 32873928

[11] Newton K. , Strasser A. , Kayagaki N. , and Dixit V. M. , Cell Death, Cell. (2024) 187, no. 2, 235–256, 10.1016/j.cell.2023.11.044. 38242081

[12] Morana O. , Wood W. , and Gregory C. D. , The Apoptosis Paradox in Cancer, International Journal of Molecular Sciences. (2022) 23, no. 3, 10.3390/ijms23031328, 35163253. PMC8836235 35163253

[13] Qin H. , Abulaiti A. , Maimaiti A. , Abulaiti Z. , Fan G. , Aili Y. , Ji W. , Wang Z. , and Wang Y. , Integrated Machine Learning Survival Framework Develops a Prognostic Model Based on Inter-Crosstalk Definition of Mitochondrial Function and Cell Death Patterns in a Large Multicenter Cohort for Lower-Grade Glioma, Journal of Translational Medicine. (2023) 21, no. 1, 10.1186/s12967-023-04468-x, 37660060. PMC10474752 37660060

[14] Carneiro B. A. and El-Deiry W. S. , Targeting Apoptosis in Cancer Therapy, Nature Reviews Clinical Oncology. (2020) 17, no. 7, 395–417, 10.1038/s41571-020-0341-y, 32203277. PMC8211386 32203277

[15] Ni L. , Yang H. , Wu X. , Zhou K. , and Wang S. , The Expression and Prognostic Value of Disulfidptosis Progress in Lung Adenocarcinoma, Aging. (2023) 15, no. 15, 7741–7759, 10.18632/aging.204938, 37552140. 37552140 PMC10457049

[16] Liu Z. , Xu X. , Liu K. , Zhang J. , Ding D. , and Fu R. , Immunogenic Cell Death in Hematological Malignancy Therapy, Advanced Science. (2023) 10, no. 13, e2207475, 10.1002/advs.202207475, 36815385. 36815385 PMC10161053

[17] Liu J. , Zhang M. , Sun Q. , Qin X. , Gao T. , Xu Y. , Han S. , Zhang Y. , and Guo Z. , Construction of a Novel MPT-Driven Necrosis-Related lncRNAs Signature for Prognosis Prediction in Laryngeal Squamous Cell Carcinoma, Environmental Science and Pollution Research. (2023) 30, no. 31, 77210–77225, 10.1007/s11356-023-26996-1, 37249774. 37249774

[18] de Souza S. , Rosario Claudio J. , Sim J. , Inyang K. E. , Dagenais A. , Monahan K. , Lee B. , Ramakrishnan H. , Parmar V. , Geron M. , Scherrer G. , Folger J. K. , and Laumet G. , Interleukin-10 Signaling in Somatosensory Neurons Controls Ccl2 Release and Inflammatory Response, Brain, Behavior, and Immunity. (2024) 116, 193–202, 10.1016/j.bbi.2023.12.013, 38081433. 38081433 PMC10843623

[19] Rylski B. , Schilling O. , and Czerny M. , Acute Aortic Dissection: Evidence, Uncertainties, and Future Therapies, European Heart Journal. (2023) 44, no. 10, 813–821, 10.1093/eurheartj/ehac757, 36540036. 36540036

[20] Li M. , Jin S. , Zhang Z. , Ma H. , and Yang X. , Interleukin-6 Facilitates Tumor Progression by Inducing Ferroptosis Resistance in Head and Neck Squamous Cell Carcinoma, Cancer Letters. (2022) 527, 28–40, 10.1016/j.canlet.2021.12.011, 34902522. 34902522

[21] Xie Q. , Wang J. , Li R. , Liu H. , Zhong Y. , Xu Q. , Ge Y. , Li C. , Sun L. , and Zhu J. , IL-6 Signaling Accelerates Iron Overload by Upregulating DMT1 in Endothelial Cells to Promote Aortic Dissection, International Journal of Biological Sciences. (2024) 20, no. 11, 4222–4237, 10.7150/ijbs.99511, 39247821. 39247821 PMC11379073

[22] Pedroza A. J. , Shad R. , Dalal A. R. , Yokoyama N. , Nakamura K. , Hiesinger W. , and Fischbein M. P. , Acute Induced Pressure Overload Rapidly Incites Thoracic Aortic Aneurysmal Smooth Muscle Cell Phenotype, Hypertension. (2022) 79, no. 4, e86–e89, 10.1161/HYPERTENSIONAHA.121.18640, 35124970. 35124970 PMC8916978

[23] Pradhan A. D. , Manson J. E. , Rifai N. , Buring J. E. , and Ridker P. M. , C-Reactive Protein, Interleukin 6, and Risk of Developing Type 2 Diabetes Mellitus, JAMA. (2001) 286, no. 3, 327–334, 10.1001/jama.286.3.327, 2-s2.0-0035908632. 11466099

[24] Jones S. A. and Jenkins B. J. , Recent Insights Into Targeting the IL-6 Cytokine Family in Inflammatory Diseases and Cancer, Nature Reviews Immunology. (2018) 18, no. 12, 773–789, 10.1038/s41577-018-0066-7, 2-s2.0-85053838588, 30254251. 30254251

[25] Kumar P. , Laurence E. , Crossman D. K. , Assimos D. G. , Murphy M. P. , and Mitchell T. , Oxalate Disrupts Monocyte and Macrophage Cellular Function via Interleukin-10 and Mitochondrial Reactive Oxygen Species (ROS) Signaling, Redox Biology. (2023) 67, 102919, 10.1016/j.redox.2023.102919, 37806112. 37806112 PMC10565874

[26] Pestka S. , Krause C. D. , Sarkar D. , Walter M. R. , Shi Y. , and Fisher P. B. , Interleukin-10 and Related Cytokines and Receptors, Annual Review of Immunology. (2004) 22, no. 1, 929–979, 10.1146/annurev.immunol.22.012703.104622, 2-s2.0-2342659126. 15032600

[27] Adam M. , Kooreman N. G. , Jagger A. , Wagenhäuser M. U. , Mehrkens D. , Wang Y. , Kayama Y. , Toyama K. , Raaz U. , Schellinger I. N. , Maegdefessel L. , Spin J. M. , Hamming J. F. , Quax P. H. A. , Baldus S. , Wu J. C. , and Tsao P. S. , Systemic Upregulation of IL-10 (Interleukin-10) Using a Nonimmunogenic Vector Reduces Growth and Rate of Dissecting Abdominal Aortic Aneurysm, Arteriosclerosis, Thrombosis, and Vascular Biology. (2018) 38, no. 8, 1796–1805, 10.1161/ATVBAHA.117.310672, 2-s2.0-85055600490, 29880489. 29880489 PMC6652227

[28] Forrer A. , Schoenrath F. , Torzewski M. , Schmid J. , Franke U. F. W. , Göbel N. , Aujesky D. , Matter C. M. , Lüscher T. F. , Mach F. , Nanchen D. , Rodondi N. , Falk V. , von Eckardstein A. , and Gawinecka J. , Novel Blood Biomarkers for a Diagnostic Workup of Acute Aortic Dissection, Diagnostics. (2021) 11, no. 4, 10.3390/diagnostics11040615. PMC8065878 33808169

[29] Saraiva M. , Vieira P. , and O′Garra A. , Biology and Therapeutic Potential of Interleukin-10, Journal of Experimental Medicine. (2019) 217, no. 1, e20190418, 10.1084/jem.20190418, 31611251. PMC7037253 31611251

[30] Wang S. , Zhang K. , Huang Q. , Meng F. , and Deng S. , TLR4 Signalling in Ischemia/Reperfusion Injury: A Promising Target for Linking Inflammation, Oxidative Stress and Programmed Cell Death to Improve Organ Transplantation Outcomes, Frontiers in Immunology. (2024) 15, 1447060, 10.3389/fimmu.2024.1447060, 39091500. 39091500 PMC11291251

[31] Sundaram B. , Pandian N. , Mall R. , Wang Y. , Sarkar R. , Kim H. J. , Malireddi R. K. S. , Karki R. , Janke L. J. , Vogel P. , and Kanneganti T. D. , NLRP12-PANoptosome Activates PANoptosis and Pathology in Response to Heme and PAMPs, Cell. (2023) 186, no. 13, 2783–2801.e20, 10.1016/j.cell.2023.05.005, 37267949. 37267949 PMC10330523

[32] Chen S. N. , Tan Y. , Xiao X. C. , Li Q. , Wu Q. , Peng Y. Y. , Ren J. , and Dong M. L. , Deletion of TLR4 Attenuates Lipopolysaccharide-Induced Acute Liver Injury by Inhibiting Inflammation and Apoptosis, Acta Pharmacologica Sinica. (2021) 42, no. 10, 1610–1619, 10.1038/s41401-020-00597-x, 33495514. 33495514 PMC8463538

[33] Wang J. , Wang J. , Meng L. , Wang X. , Yan Z. , Pan H. , Wu J. , Zhou Q. , Ye L. , Wu J. , Zhang Y. , and Wang J. , Interstitial Cystitis-Related Gene CCDC8 Accelerates Tumorigenesis by Participating in CUL7-Mediated Degradation of P53 in Bladder Cancer, Oncogene. (2026) 45, no. 8, 745–756, 10.1038/s41388-026-03688-x, 41644704. 41644704

[34] Zhang Z. , Wu M. , Yao L. , Zhou W. , Liu X. , Chen Z. , Hua P. , Xu L. , Lv L. , Liu C. , Huang C. , Chen S. , Huang Z. , Huang Y. , He J. , Chen T. , Wang J. , Yuan W. , Liu Z. , and Chen Y. , Trem2/Tyrobp Signaling Protects Against Aortic Dissection and Rupture by Inhibiting Macrophage Activation in Mice, Arteriosclerosis, Thrombosis, and Vascular Biology. (2025) 45, no. 1, 119–135, 10.1161/ATVBAHA.124.321429, 39508103. 39508103

[35] Vorkapic E. , Lundberg A. M. , Mäyränpää M. I. , Eriksson P. , and Wågsäter D. , TRIF Adaptor Signaling Is Important in Abdominal Aortic Aneurysm Formation, Atherosclerosis. (2015) 241, no. 2, 561–568, 10.1016/j.atherosclerosis.2015.06.014, 2-s2.0-84934985469, 26100679. 26100679

[36] Galluzzi L. , Guilbaud E. , Schmidt D. , Kroemer G. , and Marincola F. M. , Targeting Immunogenic Cell Stress and Death for Cancer Therapy, Nature reviews Drug Discovery. (2024) 23, no. 6, 445–460, 10.1038/s41573-024-00920-9, 38622310. 38622310 PMC11153000

[37] Zhang L. , Montesdeoca N. , Karges J. , and Xiao H. , Immunogenic Cell Death Inducing Metal Complexes for Cancer Therapy, Angewandte Chemie International Edition. (2023) 62, no. 21, e202300662, 10.1002/anie.202300662. 36807420

[38] Kroemer G. , Galassi C. , Zitvogel L. , and Galluzzi L. , Immunogenic Cell Stress and Death, Nature Immunology. (2022) 23, no. 4, 487–500, 10.1038/s41590-022-01132-2. 35145297

