# Supplementary figures and images for: IL6/IL10/TLR4 Govern Immunogenic Cell Death in Aortic Dissection
Source: Cardiovasc Ther. 2026 Mar 28;2026:4140685. doi: 10.1155/cdr/4140685 (PMC13140880; doi:10.1155/cdr/4140685)

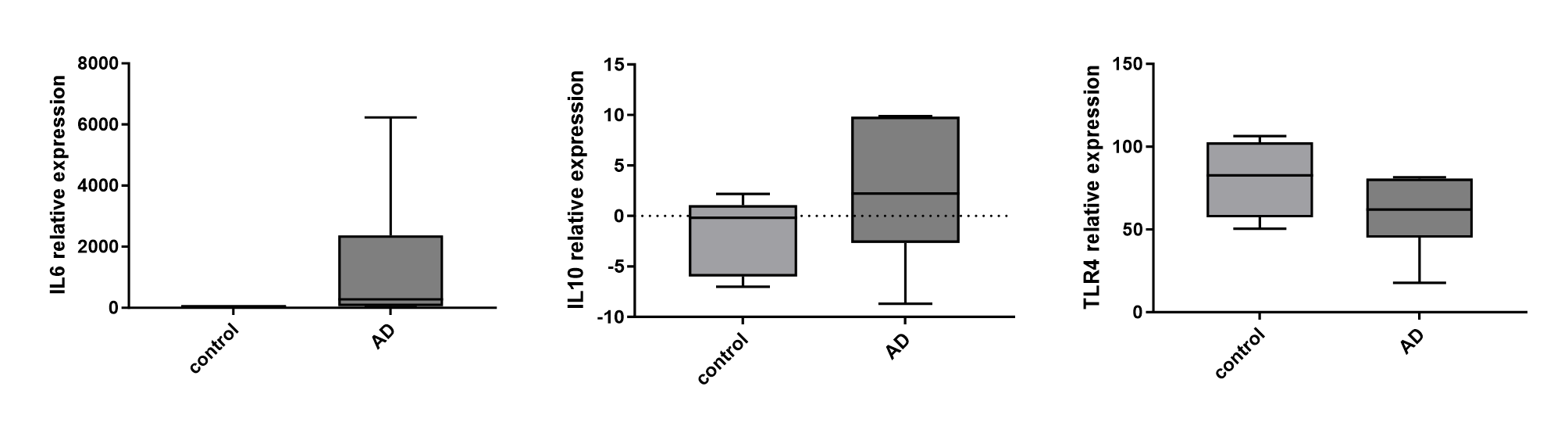

Supplement: Supplementary file 5 — Supporting Information 5 Figure S1: The expression analysis of IL6, IL10, and TLR4 in AD and controls in an independent public transcriptomic dataset (GSE52093). [file CDR-2026-4140685-s005.tif]
